# Enhanced Flexible Vacuum-Ultraviolet Photodetectors Based on Hexagonal Boron Nitride Nanosheets via Al Nanoparticles

**DOI:** 10.3390/nano16030187

**Published:** 2026-01-30

**Authors:** Youwei Chen, Jiaxing Li, Qiang Li, Wannian Fang, Haifeng Liu, Ziyan Lin, Tao Wang, Feng Yun

**Affiliations:** 1 Key Laboratory of Physical Electronics and Devices for Ministry of Education and Shaanxi Provincial Key Laboratory of Photonics & Information Technology, Xi’an Jiaotong University, Xi’an 710049, China; 202194611912@stu.xjtu.edu.cn (Y.C.); 1549159852@stu.xjtu.edu.cn (J.L.); 2 School of Electronic Science and Engineering, Xi’an Jiaotong University, Xi’an 710049, China; wannian333@stu.xjtu.edu.cn (W.F.); scorpio@stu.xjtu.edu.cn (H.L.); lzy2213525329@stu.xjtu.edu.cn (Z.L.); fyun2010@xjtu.edu.cn (F.Y.); 3 School of Physics and Astronomy, Cardiff University, Cardiff CF24 3AA, UK; wangt61@cardiff.ac.uk

**Keywords:** hexagonal boron nitride nanosheets, aluminum nanoparticles, localized surface plasmon resonance, flexible vacuum-ultraviolet photodetectors

## Abstract

This work reports an enhanced flexible vacuum-ultraviolet (VUV) photodetector on a polyimide (PI) substrate based on hexagonal boron nitride nanosheets (BNNSs) with Al nanoparticles (Al NPs). The BNNS film were prepared via liquid-phase exfoliation combined with a self-assembly process, and size-controllable Al NPs were constructed on the BNNS’s surface by electron-beam evaporation followed by thermal annealing. When the Al film thickness was 15 nm, the annealed Al NPs exhibited a pronounced enhancement of photoelectric effects at a wavelength of 185 nm. Combined with finite-difference time-domain (FDTD) simulations, it was confirmed that the localized surface plasmon resonance (LSPR) generated by Al NPs significantly enhanced the local electromagnetic field and effectively coupled into the interior of BNNSs. These exhibited a strong plasmon-enhanced absorption effect and thereby improved light absorption and carrier generation efficiency. The flexible photodetector based on this structure showed an increase in the photo-to-dark current ratio from 110.17 to 527.79 under a bias voltage of 20 V, while maintaining fast response and recovery times of 79.79 ms and 82.38 ms, respectively. In addition, the device demonstrated good stability under multiple bending angles and cyclic bending conditions, highlighting its potential applications in flexible solar-blind VUV photo ultraviolet.

## 1. Introduction

In recent years, ultraviolet (UV) photodetectors have attracted much attention due to their wide applications in space science, environmental monitoring, and flame detection [1,2,3,4,5]. Especially in the deep-ultraviolet (DUV) and vacuum-ultraviolet (VUV) areas, devices can typically achieve solar-blind detection, effectively eliminating interference from background visible and near-UV light. As a novel ultra-wide-bandgap material, hexagonal boron nitride (hBN) possesses a bandgap of approximately 6 eV, with an intrinsic absorption edge at around 207.5 nm, making it an ideal candidate for DUV and VUV photodetection. In addition, hBN exhibits excellent optical, electrical, chemical, and thermal stability, enabling reliable operation under extreme environments [6,7,8,9,10,11]. Meanwhile, its graphene-like layered structure endows it with good flexibility and adaptability to curved surfaces, allowing hBN-based flexible UV photodetectors to show great application potential in wearable electronics, flexible sensing, and nonplanar optoelectronic systems [12,13,14].

In nano-optoelectronics, the localized surface plasmon resonance (LSPR) effects have been widely used to enhance the performance of photodetectors. By exciting the plasmon resonance of metal nanostructures, the interaction between light and materials can be effectively enhanced, as well as the light absorption and Raman scattering [15,16,17]. Previous studies have shown that noble-metal nanostructures such as gold (Au), silver (Ag), and platinum (Pt) can significantly enhance photoelectric performance in the visible and near-UV regions. For example, a flexible 365 nm UV-photodetector based on Au-nanoparticle–decorated ZnO nanorods on a transparent mica substrate demonstrated a four-fold improvement in responsivity [18]. A 270 nm UV-photodetector based on amorphous Ga_2_O_3_ films with Pt nanoparticles demonstrated a 146-fold increase in responsivity [19]. However, it should be noted that noble-metal nanostructures such as Au, Ag, and Pt have achieved significant photoelectric enhancement in the visible and near-UV regions (e.g., 365 nm and 270 nm), limited by their plasmon resonance frequencies being mainly distributed in the visible to-near-UV range. When the operating wavelength is further extended toward the DUV and solar-blind region (<250 nm), noble-metal nanostructures often require complex size tuning or special structural designs to achieve effective enhancement. Their intrinsic dielectric response limitations and strong interband transition losses have restricted their application potential in DUV and VUV photodetection.

In contrast, aluminum (Al), an earth-abundant metal, possesses a higher plasmon frequency, enabling its LSPR response to naturally extend into the DUV and even VUV regions [20]. Unlike noble metals, Al readily forms a native oxide layer when exposed to ambient conditions. Upon air exposure, a few-nanometer-thick Al_2_O_3_ shell is almost instantaneously formed on the surface of Al nanoparticles (Al NPs). Previous studies have shown that Al particles with a 2–3 nm oxide layer on their surface still exhibit plasmonic response. Moreover, the oxide layer on the surface can act as a protective layer, slowing down the oxidation of the Al particles [21,22]. Recently, multiple studies have reported successful applications of Al NPs–based LSPR enhancement strategies in UV photodetector based on wide-bandgap semiconductors such as ZnO, Ga_2_O_3_, GaN, AlGaN and hBN [23,24,25,26,27,28,29,30,31,32]. For example, Kaushik et al. fabricated Al NPs on hBN surfaces via a thermal annealing method to exploit the LSPR effect. The results showed that the photocurrent was enhanced by up to 26 times at 2.5 μW cm^−2^, and the responsivity increased by approximately 60% (to 11.3 μA W^−1^) at 205 nm [32]. Although previous studies have demonstrated Al-based LSPR enhancement for deep-ultraviolet (~205 nm) photodetectors based on hBN films, the question of how to effectively introduce the LSPR effect into flexible hBN nanosheet (BNNS) films to enhance detector performance and systematically understand the underlying interaction mechanism between BNNSs and metal Al NPs remains insufficiently explored. Most reported plasmon-enhanced hBN photodetectors rely on rigid hBN structures integrated with plasmonic elements, whereas the influence of mechanical deformation on device performance has rarely been considered. Furthermore, in earlier works, plasmonic enhancement is often attributed primarily to improved light absorption, whereas the critical role of interfacial carrier transport modulation in nanosheet-assembled films has not been thoroughly investigated.

In this work, we report a flexible VUV photodetector based on self-assembled BNNS films integrated with Al NPs. The Al NPs were constructed on the BNNS’s surface via electron-beam evaporation and annealing, and their photoelectric properties were investigated. Experimental results show that the introduction of Al NPs not only significantly enhances the light absorption of BNNSs at 185 nm through the LSPR effect, but also improves carrier transport behavior through interface barrier engineering. Combined with finite-difference time-domain (FDTD) simulations, the physical mechanism by which Al NPs generate strong localized electromagnetic field enhancement and effectively couple into BNNSs in the VUV region is revealed. Furthermore, the fabricated devices exhibit stable photoelectric responses and great mechanical reliability under multiple bending angles and cyclic bending conditions, demonstrating their potential applications in next-generation flexible solar-blind UV photodetection.

## 2. Materials and Methods

**Figure 1:**
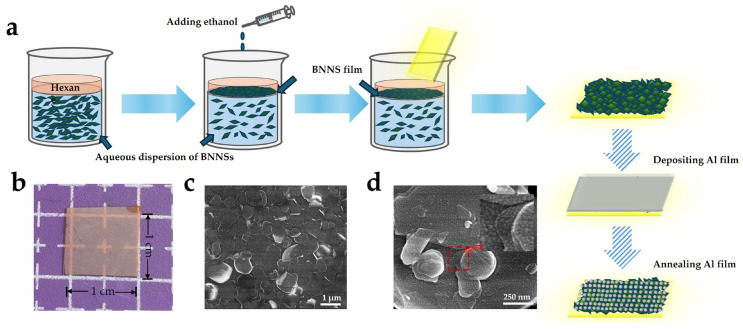
(**a**) Schematic illustration of the fabrication process of BNNS film and the formation of Al NPs via thermal annealing; (**b**) Photograph of the BNNS film on PI; SEM image of (**c**) the BNNS film and (**d**) BNNSs with Al NPs (inset shows the red area).

Relevant experiments on the preparation of BNNS films via liquid-phase exfoliation combined with self-assembly have been systematically carried out in our previous work [33]. This study focuses on transferring the self-assembled BNNS films onto flexible polyimide (PI) substrates and further constructing Al NPs on the surface through electron-beam evaporation and annealing processes to enhance photoelectric performance. Figure 1a illustrates the overall fabrication process of the device.

A Scanning electron microscopy (SEM) image (Figure 1c) shows that the BNNS film is composed of randomly stacked nanosheets with nonuniform sizes, exhibiting a typical layered polycrystalline structure. The PI substrates purchased online were sequentially ultrasonically cleaned in acetone, ethanol, and deionized water and dried with nitrogen prior to the BNNS film transfer. The thickness of the BNNS film was estimated using a surface profilometer (Dektak-XT, Bruker, Billerica, MA, USA). by measuring the step height at relatively flat regions of the film. Due to the intrinsic stacking and overlapping of nanosheets during the self-assembly process, the film surface exhibited noticeable undulations. Therefore, the measured value (~60 nm) represents the average thickness in locally smooth areas rather than a perfectly uniform geometric thickness across the entire surface. Such morphology is typical for nanosheet-assembled films and does not affect the device performance, which depends on the macroscopic conductive network formed by overlapping BNNSs. Subsequently, an Al film was deposited on the BNNS surface by electron-beam evaporation (TEMD-600, Beijing Technol Technology Co., Ltd., Beijing, China) and annealed at 350 °C for 5 min under an N_2_ atmosphere. During annealing, Al NPs with nonuniform size distribution were formed on the BNNS’s surface, as shown in Figure 1d. Notably, enrichment of Al NPs can also be observed at the edges and interfacial regions of stacked nanosheets, which provides a structural basis for subsequent interface barrier modulation. Finally, Ni electrodes were fabricated by metal-mask-assisted evaporation to construct flexible UV photodetector devices.

**Figure 2:**
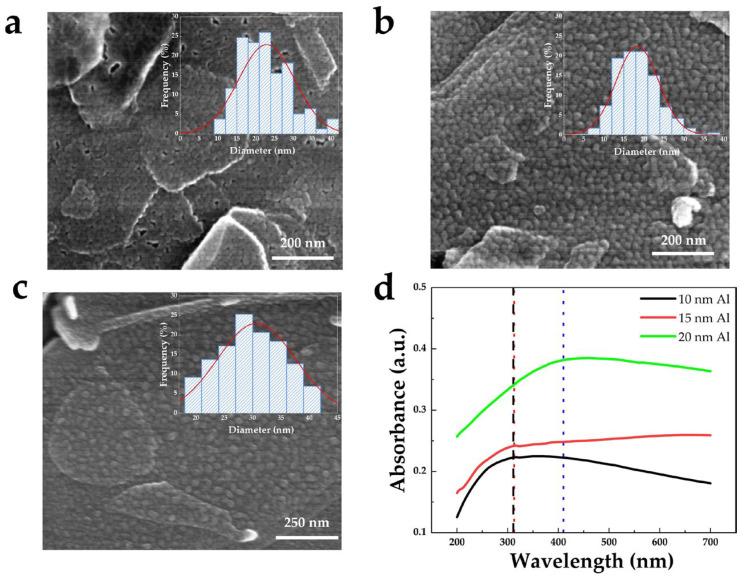
SEM images of (**a**) S10, (**b**) S15, and (**c**) S20 samples (insets: particle size distribution histograms with Gaussian fitting). (**d**) UV-visible absorption spectra of Al films with thicknesses of 10, 15, and 20 nm annealed on sapphire substrates.

To investigate the effect of Al film thickness on particle morphology and optical properties, Al films with thicknesses of 10 nm, 15 nm, and 20 nm were deposited on BNNS film and annealed under identical conditions. The samples were labeled S10, S15, and S20, respectively. Corresponding SEM images are shown in Figure 2a–c, with insets presenting statistical histograms of Al NPs size distributions and Gaussian fitting curves. The average particle diameters are 22 nm, 18 nm, and 30 nm, respectively. Comparative analysis shows that when the Al film thickness is 10 nm, the annealed Al film still exhibits partial continuity and adhesion, resulting in insufficient particle separation and a larger average particle size than that of sample S15. In contrast, sample S15 and S20 form relatively uniform nanoparticles with moderate sizes after annealing.

Figure 2d presents the UV-visible absorption spectra of the samples after annealing of Al films of different thicknesses. The annealed 20 nm Al film exhibits a pronounced absorption peak at 410.2 nm in the visible region, indicating a redshift of the plasmon resonance. In comparison, both annealed 10 nm and 15 nm Al films show absorption peaks at 312.5 nm in the UV region, with the annealed 15 nm Al film sample exhibiting significantly stronger UV absorption than the annealed 10 nm Al film. Therefore, sample S15 was selected as the optimized structure for subsequent material characterization and flexible UV device performance testing.

**Figure 3:**
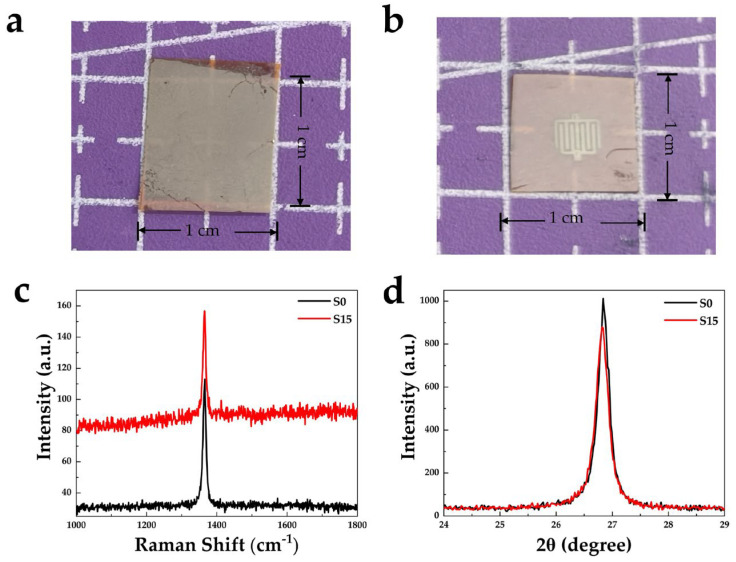
Photographs of sample S15 (**a**) before and (**b**) after deposition of 70 nm Ni electrodes. (**c**) Raman and (**d**) XRD characterization of samples S0 and S15.

A photograph of sample S15 is shown in Figure 3a. Subsequently, 70 nm thick Ni electrodes were deposited on f sample S15 by electron-beam evaporation, as shown in Figure 3b. Raman and XRD characterizations were then performed to evaluate the crystalline quality of BNNS film before and after annealing, as shown in Figure 3c,d. Raman spectra reveals that both samples exhibit the characteristic E_2_g phonon mode of hBN at 1365 cm^−1^. The full width at half maximum (FWHM) of sample S15 is 10.05 cm^−1^, slightly smaller than that of the BNNSs sample (S0), which is 11.28 cm^−1^. In addition, a significant enhancement of the Raman baseline is observed for the S15 sample compared with S0. This phenomenon may be attributed to the effective improvement of the BNNS’s surface structure induced by annealing and the interaction between Al NPs and BNNSs at the interface, which reduces defect density. Meanwhile, the formation of localized surface plasmons on the BNNS’s surface by Al NPs may amplify the Raman scattering signal, leading to the elevated baseline [34]. XRD results (Figure 3d) show that both S0 and S15 exhibit characteristic diffraction peaks of hBN at 26.84° and 26.82°, respectively, with negligible peak shift. The corresponding FWHM values are 0.28° and 0.26°, indicating that sample S15 maintains excellent hexagonal structure and high crystalline quality.

## 3. Results

**Figure 4:**
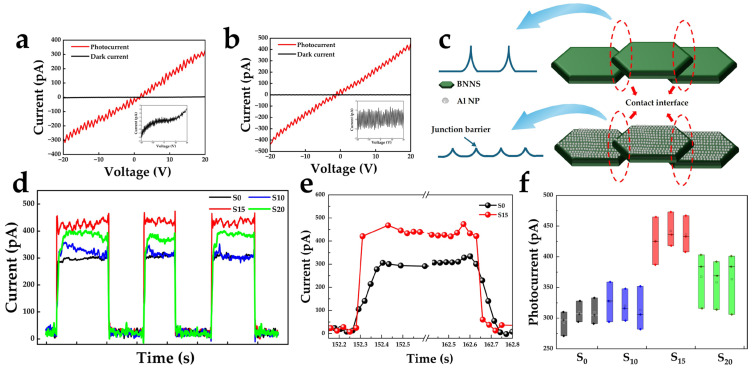
(**a**,**b**) I–V characteristics of devices S0 and S15 under dark and 185 nm illumination. (**c**) Schematic illustration of interface barrier modulation induced by Al NPs. (**d**) Temporal response characteristics curves of devices S0, S10, S15, and S20 at 20 V bias. (**e**) The response time and recovery time of devices S0 and S15. (**f**) Box plot of photocurrent distribution extracted from temporal response characteristics cycles.

Photoelectric performance tests were conducted on BNNS film and flexible devices with Al NPs. Figure 4a,b show the I–V characteristics of devices S0 and S15 under dark conditions and 185 nm UV illumination. At a bias voltage of 20 V, the dark current of device S0 is approximately 3.0 pA and the photocurrent is 330.5 pA. In contrast, the dark current of device S15 is significantly reduced to approximately 0.86 pA, while the photocurrent increases to 453.9 pA. The calculated photo-to-dark current ratios are 110.17 and 527.79, respectively, indicating that the introduction of Al NPs significantly improves the UV photoresponse performance of BNNS film.

Figure 4d shows the temporal response characteristics curves of devices S0, S10, S15, and S20 at a bias voltage of 20 V under 185 nm VUV illumination with an intensity of 30 μW cm^−2^. Device S15 exhibits the highest photocurrent (>400 pA), while the device S10 shows a photoresponse comparable to that of S0, indicating that insufficiently formed Al NPs result in weak plasmonic enhancement. Figure 4e presents the response time characteristics of devices S0 and S15. The response and recovery times were defined as the time required for the photocurrent to increase from 10% to 90% of its maximum value and decrease from 90% to 10%, respectively. The response/recovery times of device S0 are 104.23 ms and 107.17 ms, while those of device S15 are reduced to 79.79 ms and 82.38 ms. The response and recovery times have been effectively shortened. The responsivity is an important performance parameter of the photodetector and is mathematically given by the following formula:

(1) $$R=\frac{I_{ph}-I_{d}}{P_{opt}S},$$

where I_ph_ and I_d_ represent the photocurrent and dark current, respectively. P_opt_ represents the light-power density and S refers to the expose area. The responsivity of device S15 is calculated as 0.27 mA W^−1^, respectively.

In self-assembled BNNS films, adjacent nanosheets are connected via van der Waals interactions, inevitably forming interface barriers that dominate carrier transport and limit the overall conductivity and photocarrier collection efficiency of the film. Given that the BNNS film consists of stacked nanosheets, part of the deposited Al may access the intersheet contact regions during electron-beam evaporation. After annealing, these Al atoms form metallic nanoparticles at the interfaces, transforming the original semiconductor-semiconductor interface into a semiconductor-metal-semiconductor (SMS) structure. The introduction of the SMS structure leads to the formation of a double-barrier configuration. Although the effective barrier height at each interface is reduced due to the presence of metallic Al, the dark current remains suppressed because carrier transport under dark conditions is intrinsically limited by the low carrier concentration and the requirement to overcome two successive barriers. In contrast, under UV illumination, the increased photogenerated carrier density and photo-assisted barrier modulation significantly enhance carrier tunneling and thermionic emission across the SMS interfaces. Consequently, photocarrier transport and collection efficiency are markedly improved, resulting in a pronounced enhancement of the device photoresponse. A potential barrier naturally exists between two adjacent BNNSs due to the semiconductor–semiconductor contact. After the introduction of Al NPs, the original semiconductor–semiconductor interface is modified into contact regions. As a result, the single relatively high potential barrier between two BNNSs is effectively divided into two metal–semiconductor contact barriers with lower barrier heights, facilitating carrier transport across the nanosheet junctions, as illustrated in Figure 4c.

All I–V and temporal response characteristics measurements were repeated at least three times under identical conditions to ensure measurement reliability. The presented curves are representative results with negligible variation between measurements. Meanwhile, the photocurrent values extracted from multiple cycles were summarized using box plots, as shown in Figure 4f. The distribution range indicates excellent repeatability and stability of the devices.

**Figure 5:**
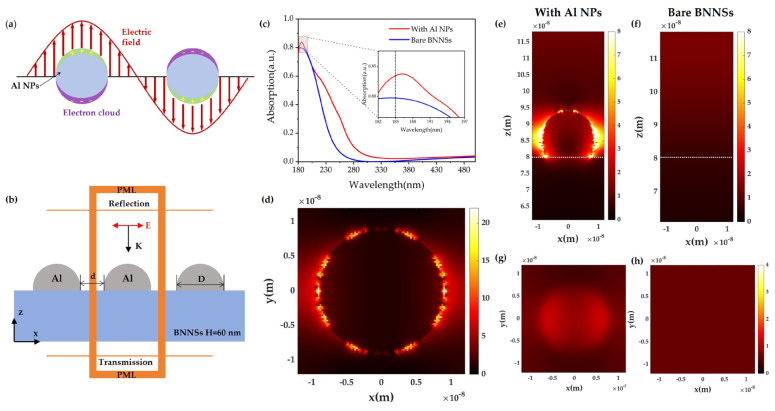
FDTD simulation of the plasmon-enhanced detection mechanism in Al NPs/BNNSs. (**a**) Schematic illustration of LSPR in Al NPs. (**b**) Simulation model of Al NPs/BNNSs, where E denotes the electric field direction, k represents the wave vector, D and d indicate the particle diameter and interparticle spacing, respectively, and PML refers to the perfectly matched layer. (**c**) Absorption spectra of Al NPs/BNNSs and BNNSs (inset: enlarged view at 185 nm). (**d**) Electric field distribution in the XY central plane of Al NPs. (**e**,**f**) Electric field distributions in the XZ plane for Al NsP/BNNSs and BNNSs, respectively. (**g**,**h**) Electric field distributions in the XY plane at the surface of Al NPs/BNNSs and BNNSs, respectively.

Localized surface plasmon (LSP) effects generally occur in metallic nanostructures when their dimensions are smaller than the wavelength of incident light. As schematically illustrated in Figure 5a, under optical excitation, the collective oscillation of free electrons on the surface of metal nanospheres leads to charge separation and the formation of a polarized electric field. In this regime, the metal nanoparticle can be treated as an oscillating dipole whose orientation follows the incident electric field. When the frequency of the incident light matches the intrinsic plasmon frequency of the met Al NPs, strong resonance occurs, resulting in a pronounced enhancement of the localized electromagnetic field near the particle surface, commonly referred to as local field enhancement (LFE).

Moreover, when two or more met Al NPs are placed in close proximity with nanometer-scale spacing, plasmonic coupling between adjacent particles gives rise to extremely confined electromagnetic fields at the interparticle gaps, known as “hot spots”. Compared to isolated nanoparticles, the electric field intensity in these hot spots can be enhanced by several orders of magnitude, significantly strengthening light–matter interactions in the surrounding medium.

In view of the experimental results, Al was selected as the plasmonic material due to its high plasmon frequency and intrinsic suitability for UV and DUV applications. To further elucidate the plasmon-enhanced UV detection mechanism, a simplified numerical model of Al-NP-modified BNNSs was established using the FDTD method, as shown in Figure 5b. In the simulation, an array of Al NPs with a diameter of 18 nm and an interparticle spacing of 6 nm was placed on top of a 60 nm thick BNNSs. A broadband plane-wave light source (180–500 nm) was normally incident on the structure. Periodic boundary conditions were applied in the X and Y directions, while perfectly matched layers (PMLs) were employed along the Z direction to eliminate spurious reflections.

The optical absorption of the structure was calculated according to the energy conservation relation:

(2) A+R+T=1,

where A, R, and T denote the absorbance, reflectance, and transmittance, respectively.

Figure 5c compares the simulated absorption spectra of pure BNNSs and Al-NP-modified BNNSs. A pronounced absorption enhancement is observed at 185 nm for the Al-NP-modified structure, whereas both structures remain nearly transparent at wavelengths above 280 nm. This result indicates that the introduction of Al NPs selectively enhances the in-band VUV absorption of BNNSs while preserving their intrinsic optical transparency in the longer-wavelength region.

To further clarify the physical origin of the enhanced absorption, the electric field distributions at 185 nm were analyzed. As shown in Figure 5d,e, strong localized electric field hot spots are generated around the Al NPs and within the interparticle gaps, arising from LSPR excitation and plasmonic coupling. In contrast, the pure BNNSs exhibit only weak and uniform electric field distributions (Figure 5f,h). Notably, the enhanced electric field penetrates into the surface region of the BNNSs at the Al/BNNSs interface, as illustrated in Figure 5g. This near-field coupling effectively extends the interaction volume between the incident light and the BNNSs, thereby significantly increasing the absorption of VUV photons.

The simulation results are in perfect agreement with the experimental observations and confirm that the plasmon-induced local field enhancement from Al NPs plays a critical role in boosting the VUV photoresponse of BNNS-based flexible photodetectors.

**Figure 6:**
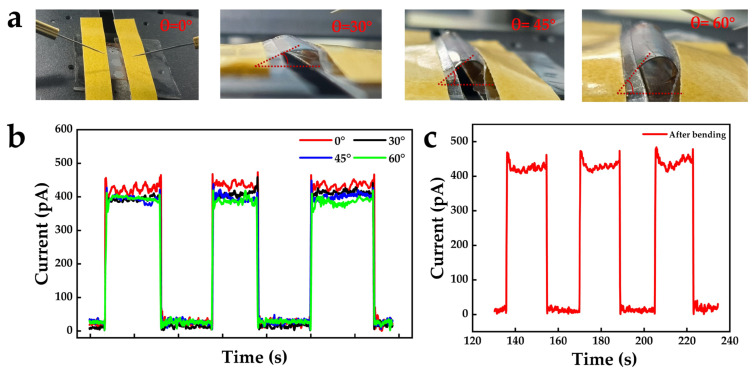
(**a**) Photographs and (**b**) temporal response characteristics curves of device S15 under different bending angles. (**c**) Temporal response characteristics after 200 bending cycles.

To evaluate the flexible stability of the device, systematic photoresponse measurements were performed on device S15 under different bending states (Figure 6a). Figure 6b shows the temporal response characteristics curves of the device at bending angles of 0°, 30°, 45°, and 60°. The results indicate that as the bending angle increases, the dark current remains nearly unchanged, while the photocurrent shows only a slight decrease and remains stable at approximately 400 pA, demonstrating good operational stability under bending conditions. The bending test was carried out by repeatedly bending the device to a fixed angle and releasing it for each measurement. Furthermore, the temporal response characteristics of device S15 were measured after 200 bending cycles at a bending angle of 60°, and no noticeable degradation in photocurrent or dark current was observed (Figure 6b), further confirming that the fabricated Al NPs/BNNS flexible VUV photodetector exhibits excellent mechanical reliability and application potential.

Finally, in Table 1, we list the performance comparisons of different plasmonic enhancement devices.

## 4. Conclusions

This work successfully constructs a flexible VUV photodetector based on Al-NP-surface-enhanced BNNSs. The results show that by rationally controlling the Al film thickness and annealing conditions, Al NPs with plasmonic responses in the VUV region can be formed on the BNNS’s surface. The introduction of Al NPs not only significantly enhances BNNSs light absorption at 185 nm via LSPR, but also improves carrier transport through interface barrier modulation, thereby increase the photocurrent to 453.9 pA and the photo-to-dark current ratio to 527.79 (4.8-fold improvement). Combined with FDTD simulations and experimental results, the synergistic physical mechanism of plasmonic enhancement is systematically revealed. In addition, the device exhibits excellent mechanical and electrical stability under multiple bending angles and cyclic bending tests. This study provides an effective strategy for the design and performance optimization of flexible VUV photodetectors.

## Figures and Tables

**Table 1:** The performance comparisons of different plasmonic enhancement devices.

Sample	Conditions	Responsivity	Response Time	Flexible	Ref.
Au Nps/ZnO	365 nm, 1 V, 3.25 mW/cm^2^	70 A/W	-	Yes	[18]
Pt Nps/Ga_2_O_3_	270 nm, 5 V, 500 μW/cm^2^	46.9 mA/W	6.9 s/7.55 s	No	[19]
Al Nps/ZnO	350 nm, 0.1 V, 0.396 mW/cm^2^	265 mA/W	-	Yes	[23]
Al Nps/GaN	355 nm, 5 V, 11 nW	670 A/W	51 ms/197 ms	No	[25]
Al Nps/AlGaN	288 nm, 5 V	288 mA/W	-	No	[26]
Al Nps/ZnO	325 nm, 5 V, 5 mW/cm^2^	267 mA/W	0.2 ms/0.4 ms	No	[27]
Al Nps/ Ga_2_O_3_	254 nm, 5 V	3.36 A/W	-	No	[30]
Al Nps/hBN	205 nm, 10 V, 2.5 μW/cm^2^	11.3 μA/W	0.5 ms/0.9 ms	No	[32]
Al Nps/BNNSs	185 nm, 20 V, 30 mW/cm^2^	0.27 mA/W	79.79 ms/82.38 ms	Yes	This work

## Data Availability

Dataset available on request from the authors.
